# PCF-Based Cavity Enhanced Spectroscopic Sensors for Simultaneous Multicomponent Trace Gas Analysis

**DOI:** 10.3390/s110201620

**Published:** 2011-01-27

**Authors:** Walter M. Nakaema, Zuo-Qiang Hao, Philipp Rohwetter, Ludger Wöste, Kamil Stelmaszczyk

**Affiliations:** Institute of Experimental Physics, Free University of Berlin, Arnimallee 14, 14195 Berlin, Germany; E-Mails: walternak@usp.br (W.M.N.); zuoqiang.hao@fu-berlin.de (Z.-Q.H.); philipp.rohwetter@fu-berlin.de (P.R.); ludger.woeste@fu-berlin.de (L.W.)

**Keywords:** molecular spectroscopy, cavity ring-down absorption spectroscopy, cavity-enhanced absorption spectroscopy, photonic crystal fibres, supercontinuum lasers, PACS 07.07.Df, 07.57.Ty, 07.60.Rd, 07.88.+y, 33.20.Kf, 42.55.Tv, 37.30.+i, 42.79.Gn

## Abstract

A multiwavelength, multicomponent CRDS gas sensor operating on the basis of a compact photonic crystal fibre supercontinuum light source has been constructed. It features a simple design encompassing one radiation source, one cavity and one detection unit (a spectrograph with a fitted ICCD camera) that are common for all wavelengths. Multicomponent detection capability of the device is demonstrated by simultaneous measurements of the absorption spectra of molecular oxygen (spin-forbidden b-X branch) and water vapor (polyads 4v, 4v + δ) in ambient atmospheric air. Issues related to multimodal cavity excitation, as well as to obtaining the best signal-to-noise ratio are discussed together with methods for their practical resolution based on operating the cavity in a “quasi continuum” mode and setting long camera gate widths, respectively. A comprehensive review of multiwavelength CRDS techniques is also given.

## 1. Introduction

Highly sensitive gas sensors that are capable to detect molecules even at low ppbv (10^−9^ Volume Mixing Ratio, VMR) or sub ppbv concentrations have recently become one of the most in-demand kinds of devices for scientific research and industrial applications. They are routinely used for tracing relevant environmental species [1–3], in medical [4,5] or clean-room diagnostics [6–8], as well as for the monitoring of hazardous or explosive materials [9,10]. Despite this remarkable versatility there are only a few analytical techniques that can offer the required levels of sensitivity; probably the most common among them are gas chromatography, mass spectrometry, and optical absorption spectroscopy.

Gas chromatography is readily used to identify the symptomatic of metabolism related diseases [11,12]. For example, untreated diabetes causes an increased concentration of exhaled acetone, while trace concentration of dimethylsulfide, methyl or ethyl mercaptane often indicate liver impairment. Methylated alkanes and benzene derivatives are known to be frequent markers of lung cancer; however, it is now clear that the effective diagnosis of this disease must take into account specific molecular profiles rather than individual compounds [13]. A multicomponent technique, like gas chromatography, is particularly well suited to accomplish this task, and a number of clinical studies have been reported in this field (see e.g., [14–16]). Besides medical applications, gas chromatographs are also used for environmental analysis [17], nonetheless, *in situ* measurements are rather seldom, most likely to due to the bulky construction of the chromatograph’s diffusion columns.

Mass spectrometry is often combined with gas chromatography constituting the so-called Gas Chromatography-Mass Spectrometry (GC-MS) technique [18]. In GC-MS a gas chromatograph separates the constituents of the sample that are subsequently ionized and detected by a mass spectrometer—typically a time-of-fight, ion trap or quadrupole (multipole) filter. The sensitivity of modern mass spectrometers is typically high and their mass accuracy parameter, *Δ(m/Z)/(m/Z)* (*m*, *Z* are molecular mass and charge, respectively), can be as small as 10^−6^. It is more than sufficient for even most demanding applications of breath analysis and allows to detect a change of mass corresponding to a single hydrogen among molecules composed of several hundred thousands of atoms. Mass spectrometers are also used as stand-alone detectors in environmental or atmospheric studies [19,20].

At present, absorption spectroscopy is probably the most straightforward, robust, and at the same time cost effective and flexible sensing technique for compound analysis. Microscopically, the effect of light absorption consists in the energy transfer from individual photons to atoms or molecules in the medium. This energy is next converted to other forms of energy, for example to heat, leading to a dissipation of the photon flux, but since the absorption spectra are always strictly related to the chemical composition of a material they can be used to identify their constituents. Inversely, if the absorbers and the absorption cross-sections are known it is possible to quantify the molecular concentrations by determining the magnitude of the radiation loss inside the sample cell (single- or multi-pass). One technique, which for this purpose uses a stable optical resonator, is known as Cavity Enhanced Absorption Spectroscopy (CEAS).

Optical resonators (optical cavities) that are suitable for the CEAS are characterized by a high quality factor (Q-factor) >10^4^ ensuring several tens of back-and-forth reflections of light during its decay and effective absorption lengths of tens of kilometres for meter-sized cavities. Thus, CEAS sensors have low detection limits and sensitivities of single pptv (VMR of 10^−12^) [21], which however, strongly depend on the reflectance of the cavity mirrors. As a consequence, the performance of these devices is very likely to degenerate in time due to the ageing process of the mirror coatings. Moreover, if the actual reflectance of the cavity mirrors is not precisely known or substantially differs from that assumed, quantitative CEAS results become uncertain. This problem is widely known and recently has been reported by Watt *et al.*, who suggested to perform additional Cavity Ring-Down Spectroscopy (CRDS) measurements in order to calibrate CEAS detectors [22].

The CRDS technique is principally distinct from CEAS, although both are sometimes classified together as cavity-enhanced techniques [23,24]. This is justified only in the sense that they both are based on high Q-factor optical cavities, but it fails to account for the fundamentally different measurement principles: in CRDS the temporal decay of the optical energy trapped in the resonator is observed, while in CEAS the absolute time-averaged intensity transmitted through it. The primary result of a CRDS measurement, the rate of intra-cavity energy loss, is expressed in terms of the so called ring-down time, *i.e.*, the decay constant of the CRDS signal. Since the difference of ring-down times determined for the empty and absorber filled cavity is directly proportional to the extinction coefficient, *α,* the CRDS is a self-referencing technique, and contrary to CEAS it is not affected by the accuracy to which the reflectance of the cavity mirrors is known; still it benefits from long absorption lengths provided by high Q-factor cavities. Initially, the CRDS was developed as a reflectometric tool for the characterization of low-loss, highly reflective mirror coatings [25]. Shortly after it was further adapted to measure absorption spectra [26]

Cavity-enhanced methods are appropriate for single- or multi-wavelength analysis of absorbers and recent advancement in the design and manufacturing of Photonic Crystal Fibres (PCFs) and the development of supercontinuum (SC) generation technologies revitalized the interest in multispectral techniques. Today, PCF-based SC light sources are readily used in CEAS [21,22,27–29], but surprisingly, they are not so popular in CRDS. To bridge this gap we have developed a multiwavelength CRDS gas sensor, which to the best of our knowledge for the first time incorporates a miniature PCF supercontinuum laser.

## 2. Broadband CRDS Techniques

A distinction between monochromatic and broadband CRDS is somewhat arbitrary and usually refers to the optical bandwidth of the incident light. The former typically uses narrow line lasers (preferably continuous wave, CW) that are spectrally and spatially matched to excite only the fundamental mode of the cavity (TEM_00_). A single-mode exponential decay is strictly monotone, which facilitates the evaluation of decay constants by reducing errors related to the fit, and which has a positive impact on the sensor’s sensitivity. But even in a single-wavelength measurement it is possible to gain knowledge about absorption spectra, yet in a stepwise manner, if the laser’s wavelength can be scanned over a line or a band of interest [26,30–33]. This scheme is called the “wavelength scan method” [24].

The broadband approach employs a different principle. Here, the light source has a sufficiently broad spectrum to cover most of the characteristic spectral features of the investigated absorber. The ring-down events are simultaneously recorded for more than one wavelength using a wavelength and time resolving detector. Thus, broadband sensors are preferable for analytical applications as they provide a fast means to discriminate between different molecular spectra. The tradeoff is the excitation of many cavity modes (longitudinal and transversal) resulting in multi-exponential ring-down decay [34]. Furthermore, interference between modes usually causes characteristic oscillations of CRDS signals [35]; however, there are also adequate methods that allow to reduce the impact of those less desirable side effects of multimode operation of the cavity. We will describe some of them later on.

Broadband techniques can be divided in two main groups. The first one constitutes a direct analogue of the wavelength scan principle with the exception that the selection of wavelengths takes place not on the side of the light source, but on the detector’s side, where the radiation leaking out of the cavity is filtered by a monochromator or a tuneable narrow line interference filter. Many authors have successfully applied this procedure, for example, Crosson *et al.* [36], who used broadband radiation from a free electron laser (25 nm FWHM centred at 5.38 μm) to measure mid-IR spectra of water vapour at a spectral resolution of 0.03 nm, or Thorpe *et al.* [37] who investigated gas phase absorption spectra of several molecules (H_2_O, O_2_, NH_3_ and C_2_H_2_) with a frequency-comb system. A distinctive feature of this device was its exceptionally broad spectral width of nearly 100 nm (at 0.5 nm wavelength resolution). Fourier transform and Fourier transform phase-shift methods constitute a separate subgroup of wavelength selective CRDS. The feasibility of Fourier transform CRDS was demonstrated for the first time by Engeln and Meijer [38] who, using broadband fluorescence radiation from a dye laser (emission spectral width 400 cm^−1^), measured a rotationally resolved spectrum of molecular oxygen between 760 and 768 nm. Several years later, Hamers *et al.* [39] used the same absorption band to demonstrate the principle of phase-shift CRDS. In this study the molecules were excited by the spectrally broad white-light from a Xenon arc lamp. However, to improve the contrast of the recorded interferograms a narrowband transmission filter (4 nm FWHM at 764 nm) had to be used, greatly reducing the actual bandwidth covered by the measurement.

A second group of broadband cavity-based methods comprise simultaneous time-wavelength techniques that are sometimes called two-dimensional [24]. Conceptually, they are very simple: broadband incident light initiates multi-wavelength ring-down, and a suitable array of photodetectors registers time-dependent, spectrally resolved intensity changes of light leaking out of the cavity—the challenge here is purely technical and mainly related to the detector. Namely, currently there are not so many detectors that would provide time resolution and wavelength discrimination simultaneously. Photomultipliers or photodiodes are sufficiently fast to measure CRDS signals of approximately μs duration, but currently their number available in an array is limited. Additionally, the area of these detectors is typically too large (∼0.5 mm × 0.5 mm) to provide a satisfactory wavelength resolution when they are attached to commercially available spectrographs with linear dispersions matched to commonly used array detectors. The elements of CMOS sensor arrays are small, but as typical day-time image sensors they are not well suited for low-intensity light applications. Charge Coupled Device (CCD) arrays are sensitive and have small pixel sizes (∼10 × 10 μm), but since they provide at best μs readout times of individual pixels (ms readout times of a full chip), they are too slow to capture CRDS signals if operated in one of the conventional exposure/readout modes.

The first CRDS technique that successfully coped with these issues—Ring-Down Spectral Photography—incorporated a streak camera principle [40,41]. The cavity outgoing light was spectrally resolved along one coordinate of the CCD detector by a grating spectrograph, and additionally deflected by a rotating mirror, scanning the dispersed light along the other coordinate such that its x- and y- directions corresponded to time and wavelength coordinates, and “spectral snap-shots” of the ring-down decay were taken in each detector row. The same approach was more recently adopted in frequency-comb based CRDS as an alternative method of obtaining molecular spectra [37].

Ball *et al.* [42,43] simplified the spectral photography setup by replacing the rotating mirror with a specially modified CCD camera. This device embodied an optically opaque mask situated right in front of the detector array. Its hidden part served as photo charge storage and was not exposed to the incident light. A few uppermost, uncovered pixel rows were used for actually generating photocharge, which was sequentially shifted by from the illuminated to the dark region of the detector. In each so called “clocking” event the charge was transferred to the next empty hidden pixel row, encoding the temporal evolution of the signal by the amount of charge contained in each row. At the end of this sequence the total charge was read row by row via the shift-register, and the CCD array was charge-cleaned for the next registration. The charge transfer method was not explicitly fast, and, as admitted by the authors, provided typical time resolution of 5 μs, only a factor 10 shorter than the average duration of CRDS signals [42].

Cavity Ring-Down Spectrography (CRD-Spectrography) bases on a simpler principle and does not require any custom-made detectors. Here, the multi-wavelength ring-down is reconstructed from a set of cavity transmitted spectra registered as independent exposures at successive, discrete delays with respect to the injected light pulse. This however requires that for every such exposure a new cavity decay has to be initiated. In addition, spectra corresponding to a particular delay are typically averaged over many laser shots to reduce noise. This procedure can be automated and is not labour-intensive, but it is inherently sensitive to any pulse energy drift of the light source, particularly if the time required for the spectrum acquisition is longer than the characteristic drift time; however, the number of time-sampled spectra and consequently the duration of the measurement is directly controllable by the number of steps in the delay sequence. The first reported CRD-Spectrography measurements on a real world sample—NO_2_– gas have been performed by Czyżewski *et al.* [44]. Their spectrograph operated on amplified fluorescence of stilbene 3 dye delivering 15 nm FWHM spectral bandwidth between 410−440 nm. This broadband radiation was passed trough the ring-down cavity and dispersed in wavelength by a spectrograph. A time evolution of the spectra was reconstructed from successive acquisitions using an intensifier fitted CCD camera (ICCD) that registered ring-down events at well defined delays with respect to the laser pulse. The sensitivity of this setup was rather modest ∼10^−5^ cm^−1^ and, as admitted by the authors, was most likely attributable to relatively low reflectivity of cavity mirrors; between 99.0% and 99.8% in selected wavelength range.

Two-dimensional CRDS setups as they were briefly described above and in general the majority of two-dimensional setups use dye-lasers to generate spectrally broad light for the cavity excitation. They are capable to deliver sub-MW optical powers typically required in broadband measurements due to the low absolute transmission of optical cavities having effective transmission (input and output mirrors) coefficients *T*∼10^−6^ or less. Lack of radiation sources being: (i) spectrally broad and (ii) rich in spectral power density (optical power per unit wavelength) has been limiting multiwavelenght CRDS for a long lime. The situation has changed with the discovery of SC light which is generated when the spectrum of a laser pulse of typically picosecond or femtosecond duration is broadened due to its nonlinear interaction with the medium in which it propagates. The short duration of the pulse ensures that MW optical powers can be generated at moderate energies of several μJ or even nJ. Not surprising is therefore that the first SC-CRDS sensor entirely based on the short pump pulse SC technology. The sensor was constructed by the group of Stelmaszczyk *et al.* and since then the technique is known as a Supercontinuum CRDS (SC-CRDS) [45].

In the innovative approach of Stelmaszczyk *et al.* [45] the SC was obtained via multiple filamentation of the beam [47] by transmitting a train of fs laser pulses (800 nm wavelength) through a block made of fused silica. This type of material is highly non-linear yielding efficient SC generation [46]. When injected into an optical cavity the SC initiates multi-wavelength light ring-down characterized by wavelength dependent decay constants. Exactly this principle was employed by Stelmaszczyk *et al.* to measure the wavelength dependent reflectance of UV CRDS mirrors. Yet, these measurements were not strictly two-dimensional basing on the wavelength-scan principle. Therefore, the group aimed at demonstrating full two-dimensional capabilities of CRDS in a subsequent experiment by measuring the absorption spectrum (423−455 nm) of NO_2_ gas [48]. The setup implemented in this case based on a CRD-Spectrography scheme and reached a detection limit of an absorption coefficient α∼6.2 × 10^−8^ cm^−1^, corresponding to 5 ppbv of NO_2_ when taking into account the average absorption cross-section of this molecule in the respective wavelength range.

More recently, the Supercontinuum Cavity Ring-Down Spectrography (SC-CRD-Spectrography) has been proposed as a broadband diagnostic tool for highly reflective mirror coatings [49,50]. Although this idea is essentially not new and, as mentioned above, has been already demonstrated by earlier investigations of Stelmaszczyk *et al.* [45,48], the new aspect of the work [49,50] is the SC light source itself, because the authors for the first time use SC radiation generated in a PCF. Nonetheless, it has never been proved whether or not such PCF-based supercontinuum sources are also eligible in ring-down setups that are designed to detect molecular absorption. First measurements with the system described below clearly confirmed such possibility.

## 3. Construction of the Broadband SC-CRDS Sensor

### 3.1. Setup

**Figure 1.**
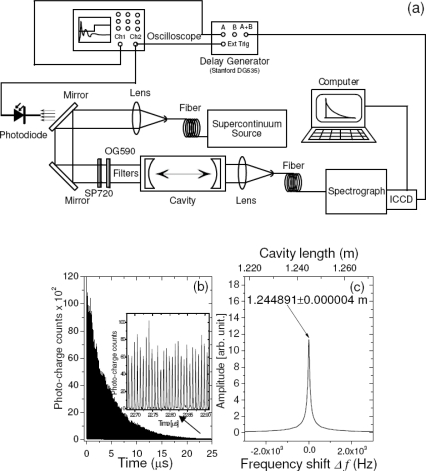
**(a)** Experimental setup. **(b)** Time dependent transmission of the cavity. Note discrete photon bursts at multiples of the round-trip time (approx. 8 ns for the 1.2 m long cavity) indicated by the inset of figure. **(c)** The power spectrum of the signal from the panel (b). Parameter *Δf* describes the frequency shift with respect to the central frequency of 120,409,796.5 Hz. The arrow indicates the optical cavity length including the effect of the refractive index of air.

**Figure 2.**
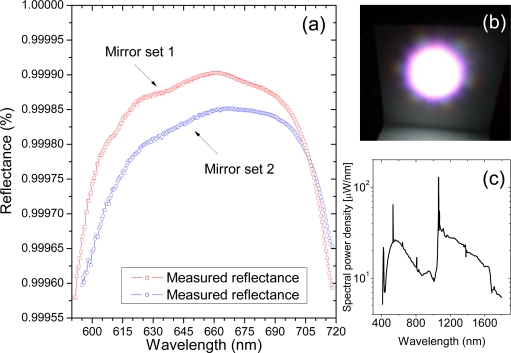
**(a)** The reflectance of cavity mirrors as measured by the setup depicted in the Figure 1. **(b)** Digital camera image of the PCF supercontinuum beam profile from the single mode PCF projected onto a screen (LP01 mode). **(c)** Spectral power density of the white light SC.

Our broadband CRDS sensor is schematically depicted in Figure 1. It incorporates the PCF supercontinuum source and the multispectral detection unit. The optical resonator encompasses two on-axis spherical mirrors (radius of curvature 6 m) spaced by a distance of approximately 1.2 m [Figure 1(c)]. Their non-confocal arrangement ensures so called “continuum” operation of the cavity. Two sets of mirrors can be interchangeably installed in the resonator. They differ slightly by the magnitude of the reflectance coefficient (Figure 2) and are suitable for measurements in VIS spectral range (600−720 nm). The SC source (Leukos Systems, SP) is a turnkey device consisting of a 1.0 m long PCF, which is optically pumped by a passively Q-switched Nd:YAG-laser. The laser may operate at variable repetition rates (1−20 kHz) and the energy and duration of its pulse are, respectively, 2.5 μJ and <1 ns. At the output of the PCF the SC beam is collimated by an aspherical lens (Ø = 25 mm, f = 20 mm, Asphericon) and two right-angle bending mirrors provide a convenient way of delivering of light into the cavity. An interference filter (Omega 3rd Millennium®, SP720) and a coloured glass filter (Schott, OG 590) are installed in front of the cavity to cut off wavelengths of the incident SC outside the block band of mirror coatings. This unwanted light would otherwise cause stray reflections inside the spectrograph degrading its performance. The cavity transmitted light is focused by a plano-convex spherical lens (f = 30 mm) onto the input of a circular-to-rectangular fibre bundle. Its output serves as the entrance slit for a f = 257 mm Czerny-Turner spectrograph (Oriel–Newport, MS257).

Two diffraction gratings were alternatively used in the spectrograph: 600 grooves/mm and 1,800 grooves/mm. The former, typically used for measurements of the reflectance of the cavity mirrors [Figure 2(a)], was offering low overall wavelength resolution and high simultaneously acquired bandwidth, respectively, 1.4 nm and 170 nm. The latter, best suited for the measurement of the molecular absorption spectra (Figure 7) enabled high wavelength resolution, but at lower optical bandwidth, respectively, 0.5 nm and 56 nm.

A gated ICCD camera (Princeton, PI-MAX with GEN III intensifier and 16-bit, 256 × 1,024 full frame, 26 × 26 μm^2^ pixel sensor equivalent to 6.6 × 26.6 mm^2^) attached to the spectrograph registers spectrally resolved cavity light. It is triggered by a TTL pulse derived from the signal of a photodiode situated behind the first bending mirror. It is delayed by 10 μs with respect to the photodiode pulse using a delay generator (Stanford, DG535), protecting the sensor against overexposure with stray light leaking through the input filter bank and transmitted by the cavity mirrors outside their specified reflectivity range. This light is initially strong, but decays at much faster rate due to lower values of the reflection coefficient. Similar problems seem common for other SC-CRDS spectrographs [49].

The trigger of the camera sets the “zero delay” of the experiment, and successive gate delays for the data acquisition are generated internally by the camera control unit. Typically, 50 gate delays (signal samples) spaced by the interval of 1.8 μs (corresponding to 0.6 MHz sampling rate) are required to time-sample one broadband CRDS transient. The ICCD control unit is also used to fine-tune the integration time of the intensifier (camera gate width). In general, this parameter is set significantly longer than the ring-down time to improve the signal-to-noise ratio (see chapter *Noise Reduction via Gate Widths*). The set of obtained cavity transmitted spectra is sent via PCI serial interface to a personal computer and stored on its hard drive.

### 3.2. Supercontinuum Light Source

A distinctive feature of our SC-CRD spectrography setup is its light source. It has a spectrum spanning over roughly 1.4 μm (400−1,800 nm) [Figure 1(c)] and the spatial mode of the outgoing light has a nearly Gaussian profile [Figure 1(b)] resulting from the fundamental propagation mode (LP01) of radiation inside the fibre. Until recently, there has not been any laser source of such a high bandwidth that is comparable only to that of thermal light. Regrettably, the spectral power density of thermal light from conventional sources like xenon flash lamps is typically low and they cannot serve well in CRDS. Thus, pulsed SC radiation generated using PCF technology [51] is much better suited for this technique.

The optical properties of a PCF are mainly defined by the geometrical distribution of air-filled holes around its core. The photonic crystal not only guides and radially confines the light but also determines the dispersive properties of the fibre, offering a high degree of attainable dispersion control. By appropriate design, fibres with multiple zero-dispersion-wavelengths may be produced that, due to a dominant role played by dispersion in SC generation, are particularly convenient for efficient white-light generation in the UV and near-IR [52,53]. Highly-nonlinear PCFs possess other interesting properties, as they are characterized by tightly confined propagation mode enhancing nonlinear response [54] of the material. In such wave guides SC is generated at comparatively low incident powers opening way for the white-light generation using pulsed ns [55,56] or even CW lasers [57]. It is worth mentioning that irrespective of the pulse duration the emitted SC has a continuous spectrum that is free from any mode structure, as it results mainly form instantaneous four-wave mixing (fs and ps time regime) or time-delayed Raman cascades (ns and CW regime) [58].

Since some spectral components of the SC are always on resonance with cavity modes (longitudinal or transverse) coupling of this light into the optical resonator demands no particular effort. This makes SC-CRDS principally easier to perform as compared to mode matched techniques. In frequency comb CRDS, for instance, the mode structure of the comb must be carefully adjusted to the mode structure of the cavity and stay unchanged for a time at least as long as one ring-down event. Thus, the comb and the cavity are typically coupled via a phase locked feedback-loops or by rapid dithering of the comb or cavity modes across one another [59].

### 3.3. Cavity

A broadband cavity should be characterized by a stable arrangement. For example, two spherical mirrors of radius of curvature *r* must be separated by a distance *d* satisfying the relation 0 < *d* < 2*r*. Additionally, the cavity layout should be non-confocal, *i.e.*, *d* ≠ *r* to accept off-axis radiation propagating as transverse modes of higher order (TEM*_n,m_*, *n,m* > 0). Their optical frequencies fit into the sparse structure of the free spectral range making the cavity transparent for arbitrary wavelengths [60]. Taking into account all these restrictions, the stability condition can be expressed by a simple inequality, 0 < *d* < *r*. The outgoing cavity light must not be clipped or blocked. It should be focused without any loss onto a detector to prevent mode selection caused by mechanical obstacles or imperfect alignment. This practice is well known from off-axis CRDS [61,62].

A flat and spectrally broad reflectance characteristic of the mirror coatings is in general desirable as it ascertains a time-balanced decay of many spectral components and similar dynamic range of their signals. However, reasonable variations of the reflection coefficient will not primarily be harmful because in CRDS this parameter does not contribute in the determination of the absorption coefficient. On the other hand a narrow bandwidth of cavity mirrors always restricts broadband measurements even if the limitations are purely technological and result from the density of multilayer dielectric coatings. For example, the bandwidth of recently available UV coatings is typically limited to 20−30 nm, while that of VIS IR coatings can reach 100−200 nm [63]. At present the limited bandwidth of high reflectance of cavity mirrors is one of the most important barriers for achieving exceptionally broadband measurements.

### 3.4. Performance of the Optical Resonator

**Figure 3.**
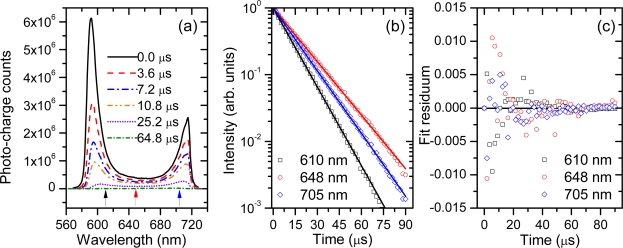
**(a)** The transmission of the cavity measured at different time delays (indicated above the transmission curves) with respect to the camera trigger. **(b)** Measured and fitted ring-down signals for the wavelengths that are indicated by arrows situated below the transmission curves on panel (a). The decay constants are *τ_0_* = 11.127 ± 0.026 μs, *τ_0_* = 16.097 ± 0.075 μs, *τ_0_* = 13.882 ± 0.046 μs, respectively, from the shortest to the longest of wavelengths. **(c)** Fit residua of the signals from panel (b).

An example of typical cavity-transmitted spectra and the resulting multi-wavelength CRDS signals, obtained with the evacuated resonator is shown in Figure 3. The wavelength interval covered by one channel (pixel) in the presented case is 0.17 nm. Within this spectral bandwidth several thousands of longitudinal cavity modes were excited, because in the respective wavelength interval and for 1.2 m long cavity the density of modes is ∼10^4^ nm^−1^. If excited, these modes co-propagate in the cavity and mode beating effect may be observed. But since such beating occurs at the free spectral range (125 MHz for our cavity) or its harmonics it cannot be resolved at much lower sampling rate of the signal (in our case 0.6 MHz). Indeed, the high frequency beating is not seen on the CRDS signals [Figure 3(b)], as confirmed by the residua of the fitted functions presented on the Figure 3(c).

**Figure 4.**
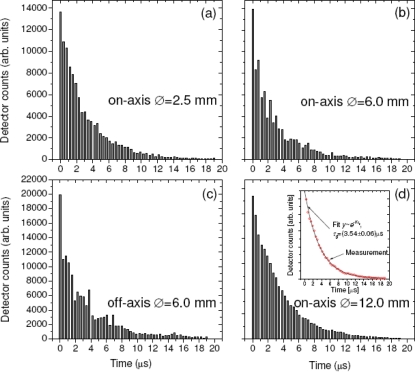
The effect of the transverse modes beating within 13 nm optical bandwidth (720–733 nm) for various scenarios of cavity excitation. **(a)**, **(b)**, **(d)** on- and **(c)** off-axis excitation. Inset of figure **(d)** additionally shows single exponential curve fitted to the data. Note different diameters of the exciting beam that are indicated on each of the panels.

In practice, however, the remaining oscillations of CRDS signals cannot be fully removed and are typically caused by transverse cavity modes (TEM*_n,m_*, where *n,m* > 0) that interfere at much lower frequencies. Their beating becomes particularly relevant if the beating period is close to the decay constants [64]. The effect of the transverse mode beating and its reduction is portrayed on the graphs from the Figure 4. In each case the cavity was excited by the collimated SC beam, which illuminated only selectively chosen parts of the input mirror area. The two upper graphs and the bottom right graph [Figure 4(a,b,d)] correspond to the cases of excitation in which the cavity axis and the incident beam axis coincide; panel 4c corresponds to off-axis excitation when the beam axis was shifted with respect to optical axis of the cavity by 6 mm. The diameter of the beam is, respectively, 2.5 mm, 6.0 mm and 12 mm for panels 4a, 4(b,c) and 4d. The largest of diameters corresponds to the clear aperture of the input mirror.

Evidently, the beating among transverse modes is largely reduced if the incident beam is on-axis and covers just a small area around the optical axis [Figure 4(a)]. It is consistent with the fact in this configuration the probability of activating higher order transverse modes is low, as they occupy the space further away from the resonator’s axis resulting in a poor spatial overlap with the small diameter incident beam. On the contrary, large beams intercepting a larger area around the axis excite transverse modes with much higher probability giving rise to apparent mode beating [Figure 4(b)]. The effect is further enhanced during off-axis excitation [Figure 4(c)] in which case the longitudinal modes are not excited directly, but via ineffective internal mode coupling [34]. As a consequence, the number of active transverse modes is meaningfully larger than the number of active longitudinal modes.

The last of the graphs [Figure 4(d)] depicts apparently different operation regime of the broadband cavity. Here, a large number of simultaneously excited transverse and longitudinal modes establish a “quasi-continuum”, in which virtually all wavelengths of the injected SC undergo ring-down decays. The phase of individual cavity modes cancels out and their beating becomes negligible, as confirmed by the weak signal oscillation seen in Figure 4(d). It is important to understand that (besides a non-confocal cavity arrangement) it is necessary for the incident SC beam to fill the largest possible area of the input mirror and excite as many cavity modes as possible. Therefore, the criterion suggested by Schmidl *et al.* “to achieve the smallest possible diameter of the propagating beam on the mirrors” may not be adequate for eliminating the effect of transverse mode beating. In this context it is not surprising that characteristic “waving” appears on the signals presented in their work (see e.g., Figure 2(b) in [49] and other therein). On the contrary, the quasi-continuum light decay is to large extent similar to the *Beer-Lambert*-like decay (see the inset of the graph (d)). Therefore, well designed SC-CRDS cavities should be working in this regime.

### 3.5. Noise Reduction via Gate Widths

In our previous work we have suggested to use long camera gate widths for the acquisition of SC-CRD-Sprctrography signals [48]. Consequently, each of the spectra from Figure 3 was obtained by integrating the cavity-transmitted light over 50 μs long intervals representing roughly a half-time of the total signal duration [Figure 3(b,c)]. At first glance the procedure seems counterproductive, because in the most straightforward approach a time dependent (photo) signal is typically sampled at time intervals that are much shorter than its characteristic temporal variation. Nonetheless, it is easy to show why it is possible to apply such long gate widths in CRD-Spectrography.

A photo signal *s* representing monochromatic ring-down decay at any time *t > 0* can be written as:

(1) $$s(t)=I_{0}e^{-\;\frac{t}{\tau_{0}}},$$

where *I_0_* denotes the intensity transmitted by the cavity at *t* = 0 (initial intensity) and *τ_0_* denotes the ring-down time. Equation 1 applies to any signal acquired with a detector (e.g., photodiodes or photomultipliers) of response time that is much shorter as compared to *τ_0_*. In SC-CRD-Spectrography the situation can be quite different, because the photo charge is collected at discrete camera gate delays, *t_i_*, and within a certain time interval determined by the duration of the gate width, *τ_g_*, which—as noted earlier—may be meaningfully longer than *τ_0_*. Consequently, the ring-down signal must be represented by the integral of *s(t)* over the time interval defined by *t_i_* and *t_i_+τ_g_*, *i.e.*,:

(2) $$S(t_{i},\tau_{g})=\int_{t_{i}}^{t_{i}+\tau_{g}}s(t)\;dt=I_{0}\tau_{0}\left(1-e^{-\;\frac{\tau_{g}}{\tau_{0}}}\right)e^{-\;\frac{t_{i}}{\tau_{0}}},$$

(3) $$\underset{\tau_{g}>>\tau_{0}}{\text{lim}}S(t_{i},\tau_{g})=I_{0}\tau_{0}e^{-\;\frac{t_{i}}{\tau_{0}}}.$$

Direct substitution of *t_i_* by *t* in Equation 2 proves that the ring-down times given in Equation 1 and Equation 2 are strictly the same and either of the equations can be equivalently used to derive *τ_0_*. Nonetheless, the integrated signal has one eminent feature, namely, that its amplitude inherently depends on the camera gate width *τ_g_*. Generally speaking, longer gate widths yield more light during the exposure and higher values of ∫ *s*(*t*)*dt* improving an overall signal-to-noise ratios due to improved photon statistics and more accurate analogue-to-digital conversion; the maximum is approached when *τ_g_* *>> τ_0_* (Equation 3).

**Figure 5.**
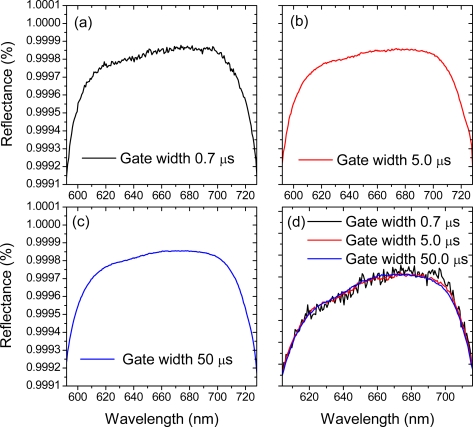
Reflectance of the mirrors of set 2 derived from CRD-Spectrography signals obtained by applying ICCD camera gates of different durations: **(a)** *τ_g_* = 0.7 μs, **(b)** *τ_g_* = 5.0 μs, **(c)** *τ_g_* = 50 μs. **(d)** Reflectivity curves superimposed one on another. Note the reduction of fluctuations with increasing gate width. The scale is not compatible with this of the other panels and was changed to emphasize the role of the gate duration.

The possibility of using the duration of the gate width for improving quality of SC-CRDS signals has been recently questioned by Schmidl *et al.* [49], who supposed that longer gate widths cause more noise (presumably electromagnetic) affecting the acquired data. But since integral techniques have widespread and successful applications in the signal acquisition equipment and are readily used, for example, in boxcar integrators [65] we performed a series of simple test measurements to check their applicability in broadband CRDS. Figure 5 illustrates the result. Each of the curves from this figure represents once again the measured reflectance of the cavity mirrors (mirror set 2). The data was acquired under similar experimental conditions: similar intensity and repetition rate of SC pulses, the same signal averaging and signal wave-form length *etc.* and the only varied parameter was the camera gate width *τ_g_*. The quality of the obtained curves visibly improves with increasing duration of *τ_g_*. It is a direct consequence of the signal integration as indicated by Equations 2 and 3.

It is appropriate to point out that our proposed method of signal integration is best suited for removing not only (i) random background noise (thermal and electronic) but also (ii) improves the photon statistics, which may become a dominant cause of signal fluctuations in the single photon regime when CRDS transients consist of spikes of strongly varying amplitude according to the Poissonian statistics. Such signals are not satisfactory well approximated by smooth exponential functions [45].

## 3. Absorption Measurements

The VIS absorption (610–730 nm) of atmospheric air at normal pressure was investigated with the SC-CRDS sensor of Figure 1. Before each experiment run the cavity was evacuated and reference decay constants were determined. The resonator was next filled with dust free air initially purged through a PTFE Teflon® syringe filter (GE Osmonics Labstore, pore size 0.45 μm) to remove bigger particles and minimize losses related to Mie scattering. The gas inside the cavity consisted of a mixture of dry atmospheric air (lab air) and humid air, which was generated by bubbling the dry air in a glass vessel containing distilled water. Depending on the rate, normally 2−6 litres per minute, the relative humidity (RH) of the humid air was typically between 50−80%. The RH of the dry air was systematically lower, in the range of 20−40%, and was mainly dependent on meteorological conditions. The dry and humid air were mixed at known partial pressures the sum of which was corresponding to the ambient pressure. Additionally, the actual value of RH inside the cavity was verified before each series of experiments with the precision of ±2% absolute using the thermohygrometer (ATP Messtechnik GmbH P470). Direct contact of the gas with stainless-steel cavity walls provided a good thermal equilibrium with the ambient temperature of 22 °C. A special precaution was taken to assure a quasi-adiabatic expansion of the air, which otherwise would lead to water condensation.

At atmospheric pressure the fraction of H_2_O molecules that is adsorbed on the cavity walls is typically negligibly small. Thus, it is reasonable to estimate the actual concentration of gas-phase water dipoles(*N*_*H*_2_*O*_) from the physical parameters of the inlet air and the formula:

(4) $$N_{H_{2}O}=\frac{p^{*}{}_{H_{2}O}}{p_{\mathrm{atm}}}\frac{V}{k_{B}T}\left(RH_{\mathrm{dry}}\;\;p_{\mathrm{dry}}+RH_{\mathrm{humid}}\;\;p_{\mathrm{humid}}\right).$$

**Figure 6.**
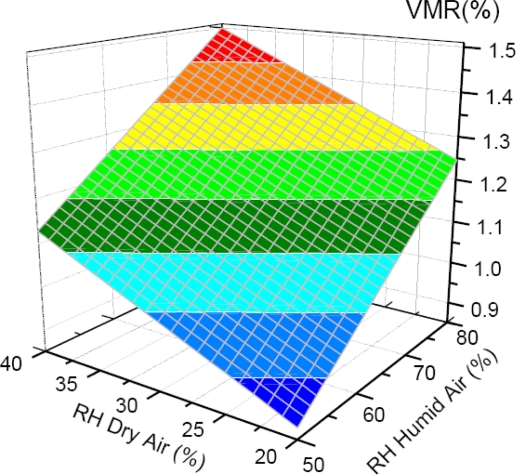
Calculated volume mixing ratio of H_2_O molecules in the cavity containing 500 hPa of moist air (513 hPa dry air) as a function of the RH of the dry and moist air.

It takes into account an ideal gas. In Equation 4 p^*^_H_2_*O*_ and *p_atm_* denote the saturation pressure of water vapour [66] and the atmospheric pressure, respectively, *V* is the volume of the cavity, *k_B_* is the Boltzmann constant, *T* is the temperature and the *RH* and *p* correspond to the relative humidity and the pressure of the dry and humid air. The result of such theoretical estimation is shown in the Figure 6. In the presented case the partial pressure of humid air is 500 hPa. The left panel corresponds to the number concentration of water molecules, while the right one to their corresponding volume mixing ratio. Both quantities are plotted as a function of the RH of the dry and humid air. According to the calculation the partial pressure of water in the cavity does not exceed 17 hPa (1.6 % VMR) and for the ambient temperature of 22 °C is well below the saturation pressure of 25.5 hPa [66]. Indeed, virtually all water molecules inside the cavity are in the gas phase.

**Figure 7.**
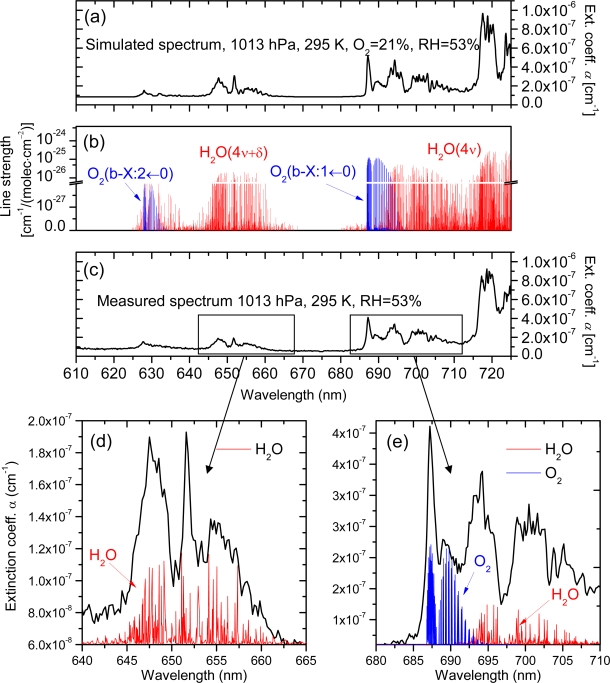
**(a)** The absorption spectrum of atmospheric air simulated using the Hitran 2000 data base. A background extinction taking into account Rayleigh scattering is added. **(b)** Line strengths with indicated molecular transitions. **(c)** The measured spectrum (mirror set 1). **(d)** and **(e)** A close-up of selected parts of the spectrum.

Figure 7(c) shows a spectrum as it was typically obtained in experiments. It covers two stretching-bending overtones of H_2_O (polyads 4ν + δ and 4ν) and two vibronic transitions of the weak spin-forbidden b-X band of O_2_ (b^1^Σg^+^*(ν″* = *1)*←X^3^Σg^−^*(ν′* = *0)* and b^1^Σg^+^*(ν″* = *2)*←X^3^Σg^−^*(ν′ = 0)*). Figure 7(b) and 7(a) present, respectively, the line strengths and simulated absorption spectrum of air for the corresponding wavelength range. For the simulation the Hitran 2000 data base [67] was used and 21% VMR of oxygen and the 53% RH were assumed the latter corresponding to the value measured in the cavity.

Since the ratios of line strengths of O_2_/H_2_O do not differ by more than 5–8% absolute, we can state relatively good agreement between measurements and simulations. Nonetheless, it is sensible to ask why it is not possible to obtain accurate quantitative information in our measurements even though the absorption cross-sections were precisely known. It turns out that the problem is more general and relates to the wavelength resolution of the measurement, which in our case was not sufficient to resolve narrow line resonances of absorbing species, because typical line widths of, O_2_ and H_2_O at normal pressure (1,013 hPa) are 7 GHz (0.01 nm) and 4 GHz (0.005 nm), respectively, far beyond the resolving capabilities even of the 1,800 grooves/nm grating. Such narrowband resonances tend to saturate over long absorption paths encountered in the cavity, and since even a weak saturation suffices to alter the spectra, they cannot be represented by a simple convolution of absorption cross-sections with the instrument slit function. The net result is that true molecular concentrations are hardly derivable unless special retrieval algorithms are used [43]. Such advanced data treatment is, however, beyond the scope of the present work, which aimed at demonstrating applicability of PCFs in CRD-Spectrography.

Finally, we point out that the magnitude of energy dissipation in the cavity that is not related with absorption losses or transmission losses through the mirrors further confirms a good quantitative performance of the sensor. It can be determined in spectral windows that are free from molecular resonances of O_2_ and H_2_O; in our case 610−625 nm and 670−680 nm. The wavelength averaged rate of the energy loss determined in this way is 7.1 ± 1.2 × 10^−8^ cm^−1^. The Rayleigh scattering theory yields a similar value of 5.9 × 10^−8^ cm^−1^ and the small discrepancy of about 1 × 10^−8^ cm^−1^ is likely caused by Mie scattering on residual dust particles. In any case, the overwhelming part of the background signal must have been caused by Rayleigh scattering.

## 4. Conclusions

We have demonstrated the suitability of a compact PCF based SC sources for the simultaneous CRDS measurement of multiple absorbing components in a broad spectral region. This simple approach offers a range of several potential applications such as humidity control, monitoring of air quality in tunnels and chimneys, detection of hazardous substances in airports *etc.*, where a fast and reliable sensor to detect changes in concentration is needed.

The stray light coming from internal reflections inside the spectrograph can considerably affect the signal, causing multi-exponential decays at the very beginning of the transients, and it also limits the dynamic range of the detector due to the initial high intensities. Thus, one should consider using gated devices to control the exposure time of the sensor, besides filters to select the spectral range of interest and reduce problems related to stray-light reflection that are common for grating spectrometers. Additionally, the reflection coefficient curve of the SC-CRDS mirrors should be possibly flat to achieve similar dynamic ranges at all wavelengths of interest.

Although the maximum resolution of the instrument is approximately one order of magnitude lower than would be necessary to resolve the absorption lines of typical atmospheric relevant molecular absorbers, it is not a limiting factor for their identification. However, accurate quantitative information on their concentration can be obtained only when special retrieval algorithms are applied, which produce a linearized absorption cross-sections throughout the fit of the natural logarithm of the time dependent cavity transmission spectra convolved with the resolution of the instrument as described in [43].
